# The role of neoadjuvant chemotherapy in patients with locally advanced colon cancer: A systematic review and meta-analysis

**DOI:** 10.3389/fonc.2022.1024345

**Published:** 2022-10-11

**Authors:** Zongyu Liang, Zhu Li, Qingshui Yang, Jiahao Feng, Deyu Xiang, Haina Lyu, Guangzhi Mai, Wanchuan Wang

**Affiliations:** Second Department of General Surgery, The Sixth Affiliated Hospital, School of Medicine, South China University of Technology, Foshan, China

**Keywords:** neoadjuvant chemotherapy, locally advanced colon cancer, overall survival, R0 resection, perioperative complication

## Abstract

**Background:**

Controversy persists about neoadjuvant chemotherapy (NAC) within the field of locally advanced colon cancer (LACC). The purpose of this study was to assess the existing and latest literature with high quality to determine the role of NAC in various aspects.

**Methods:**

A comprehensive literature search of the PubMed, Embase, Web of Science, and the Cochrane Library databases was conducted from inception to April 2022. Review Manager 5.3 was applied for meta-analyses with a random-effects model whenever possible.

**Results:**

Overall, 8 studies were included in this systematic review and meta-analysis, comprising 4 randomized controlled trials (RCTs) and 4 retrospective studies involving 40,136 participants. The 3-year overall survival (OS) (HR: 0.90, 95% CI: 0.66-1.23, P = 0.51) and 5-year OS (HR: 0.89, 95% CI: 0.53-1.03, P = 0.53) were comparable between two groups. Mortality in 30 days was found less frequent in the NAC group (OR: 0.43, 95% CI: 0.20-0.91, P = 0.03), whereas no significant differences were detected concerning other perioperative complications, R0 resection, or adverse events. In terms of subgroup analyses for RCTs, less anastomotic leak (OR: 0.51, 95% CI: 0.31-0.86, P = 0.01) and higher R0 resection rate (OR: 2.35, 95% CI: 1.04-5.32, P = 0.04) were observed in the NAC group.

**Conclusions:**

NAC is safe and feasible for patients with LACC, but no significant survival benefit could be demonstrated. The application of NAC still needs to be prudent until significant evidence supporting the oncological outcomes is presented.

**Systematic review registration:**

https://www.crd.york.ac.uk/prospero, identifier (CRD42022333306).

## Introduction

According to the report by the American Cancer Society in 2022 (1), approximately 151,030 individuals of both sexes would be newly diagnosed with colorectal cancer (CRC), of which 70.3% would be colon cancer. The prognosis of patients with CRC has been greatly improved following advances in surgical concepts and techniques. The CONCORD-2 trial (2) revealed that 5-year cancer-specific survival of localized, regional, or metastatic colon cancer was 90%, 70%, and 14% respectively in the United States. In recent years, neoadjuvant therapy has been widely and well applied in solid tumors such as mammary, esophageal, gastric, and rectal cancers. Notably, preoperative concurrent chemoradiotherapy has been considered the preferred standard treatment for locally advanced rectal cancer (3, 4), while neoadjuvant treatment remains controversial in the field of locally advanced colon cancer (LACC). The National Comprehensive Cancer Network (NCCN) Guideline for colon cancer has provided recommendations since 2016 that neoadjuvant chemotherapy (NAC) such as FOLFOX or CAPEOX could be an alternative primary treatment for clinical T4b findings, aiming to improve R0 resection rate, postoperative recovery, and survival outcome. On the other hand, the only two published randomized controlled trials including the FOXTROT (5) and PRODIGE 22 (6) trials focusing on survival outcomes both failed to demonstrate the oncological benefit of NAC in patients with LACC. Additionally, the safety and feasibility of NAC should also be cautiously assessed. Therefore, we performed this systematic review and meta-analysis of the existing and latest literature with high quality to determine the role of NAC in patients with LACC.

## Material and methods

### Study selection

This systematic review and meta-analysis was performed in accordance with the Preferred Reporting Items for Systematic Reviews and Meta-Analysis (PRISMA) (7, 8) and Meta-analysis Of Observational Studies in Epidemiology (MOOSE) (9) reporting guidelines. This study has been registered in the International Prospective Register of Systematic Reviews (PROSPERO). A comprehensive literature search of the PubMed, Embase, Web of Science, and the Cochrane Library databases was conducted from inception to April 2022.

Following were the inclusion criteria for this study: (a) studies concerning patients with locally advanced colon cancer, (b) studies comparing outcomes between NAC and upfront surgery with adjuvant chemotherapy, (c) studies reporting survival outcomes, perioperative complications, adverse events of chemotherapy, or tumor characteristics on pathological examination and, (d) RCTs or cohort studies. Exclusion criteria were as follows: (a) cases with distant metastasis, (b) cases with perioperative radiotherapy or intraoperative chemotherapy, (c) studies concerning the non-human subject, (d) articles of letter, case report, review, editorial, comment or only protocol, (f) without adequate data for analysis and, (g) non-English publications.

### Data extraction and types of outcomes

Demographics extracted from included studies consisted of year, country, study design, Union for International Cancer Control (UICC) tumor stage, number of participants, age, sex, chemotherapy regimen, and chemotherapy completion.

The primary outcomes of this study assessed were survival outcomes. Secondary outcomes included perioperative complications, adverse events of chemotherapy, and tumor characteristics on pathological examination. Eventually, variables capable for meta-analyses consisted of OS, anastomotic leak, wound infection, abscess, ileus, re-operation, stoma, R0 resection, 30-day mortality, and grade 3 or higher adverse events of chemotherapy. Additionally, cases diagnosed with T3 with extramural depth ≥ 5 mm or T4 and RCTs would be respectively selected for subgroup analyses.

Two authors (Liang and Li) independently extracted and cross-checked all relevant data from included studies. In case of discrepancies, a third author (Yang) was asked to discuss until a consensus was reached.

### Quality assessment

A measurement tool for the ‘assessment of multiple systematic reviews’ (AMSTAR) (10) consisting of 11 items with good face and content validity for measuring the methodological quality of systematic reviews was used. We carefully read the original literature and the details from the clinical trials registry (https://clinicaltrials.gov/ ) if available and then, evaluated the quality of literature. The modified Jadad quality scale (11) ranging from 0-7 points was used for bias assessment of RCTs and the Newcastle-Ottawa scale (NOS) (12) ranging from 0-9 points was for non-RCTs in this systematic review, with higher scores indicating better quality. Studies scoring greater than or equal to 4 points of the modified Jadad scale or 5 points of the NOS were considered high quality and therefore eligible.

Quality assessment was rated by two review authors (Liang and Li). In case of disagreements, a third author (Yang) was asked to participate in discussion until a consensus was reached.

### Statistical analysis

The major demographic characteristics of all the included studies were summarized by a basic descriptive statistical method. Chi-squared and Fisher’s exact tests were used for categorical and continuous variables, respectively. A systematic review and meta-analysis were performed following accumulation of sufficient research data. The software Review Manager, version 5.3 (https://community.cochrane.org/help/tools-and-software/revman-5) was used to analyze the data and a random-effects model was used to calculate the pooled effect estimates. Survival outcomes were presented as hazard ratios (HRs) with 95% confidence intervals (CIs). If HRs of included studies were not reported directly, an estimated HR was derived from Kaplan-Meier curves based on the method raised by Tierney et al. (13). In addition, continuous variables were analyzed by weighted mean differences (WMDs) with 95% CI, and odds ratios (ORs) with 95% CIs were used to assess dichotomous variables. All results compared were considered statistically significant at a two-sided P < 0.05.

The heterogeneity was evaluated by the Cochrane Q test and Higgins *I* ^2^ test. A sensitivity analysis or subgroup analysis would be conducted once the heterogeneity was considered high (P < 0.1 or *I* ^2^ > 50%).

## Results

### Study selection and quality assessment

**Figure 1:**
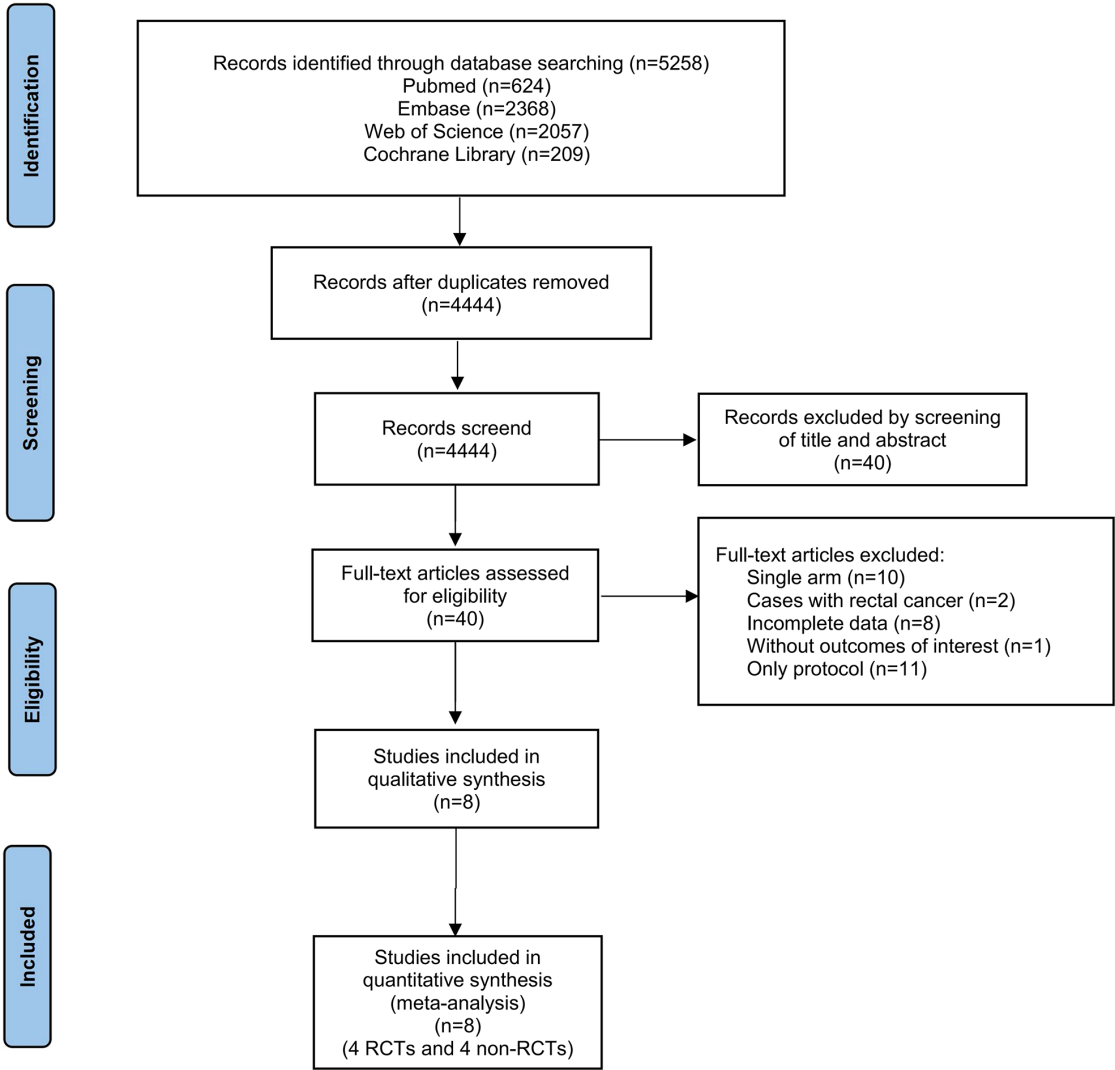
PRISMA selection flow diagram.

Our literature search yielded 4444 potential studies after duplicates were removed **(** **Supplementary Table 1-4** **)**. Of these, 40 full-text articles were considered for inclusion. Eventually, a total of 8 published studies were included in this systematic review and meta-analysis. The flow diagram of the inclusion & exclusion process was presented in **Figure 1** and also, reasons as well as references for full-text articles excluded were presented in **Supplementary Table 5**. The modified Jadad and NOS scale were used for quality assessment, shown in **Supplementary Table 6**.

### Demographic characteristics

The major demographic characteristics of all the included studies consisting of 4 RCTs (5, 6, 14, 15) and 4 retrospective research (16–19) were summarized in **Table 1**. Notably, the FOXTROT trial (14) and Morton (5) independently reported results from the same registered clinical trial but different centers. Karoui et, al reported short-term outcomes in 2020 (15) and survival outcomes in 2021 (6) among the same participants. All of the included studies were conducted in multiple centers from Europe or America. In total, 40136 patients diagnosed as T3 or T4according to UICC tumor stage were included in the pooled analysis, with 2793 (7%) undergoing NAC. Among the retrospective studies included except for Gooyer, et al. (17), NAC was more likely to be administrated to younger patients. Only RCTs recorded the regimen and completion rate of chemotherapy.

**Table 1:** Baseline characteristics of studies included.

Study	Design	Stage	Number of participants	Number of nCT (%)	Median age (range),years old		Female / Male		Chemotherapy regimen
**nCT**	**pCT**	**nCT**	**pCT**
Foxtrot, UK,2012	RCT, Multi-center	T3 with extramural depth ≥5 mm or T4	150	99 (66%)	64 (31-82)	65 (38-78)	34/65	19/32	OxMdG
Dehal, USA,2018	Retrospective, Multi-center	T3 or T4	27575	921 (33%)	58.4 (12.1)*	61.5 (12.8)*	388/533	13384/13270	NA
Morton, UK,2019	RCT, Multi-center	T3 with extramural depth ≥5 mm or T4	1053	699 (66%)	NA		NA		OxMdG
Gooyer, NLD,2020	Retrospective, Multi-center	T4	2146	192 (9%)	64 (29-84)	64 (25-88)	91/101	961/993	NA
Karoui, France,2020	RCT, Multi-center	T3 with extramural depth ≥5 mm or T4	104	52 (50%)	65 (46-79)	62 (30-75)	22/30	19/33	simplified FOLFOX-4
Karoui, France,2021	RCT, Multi-center	T3 with extramural depth ≥5 mm or T4	104	52 (50%)	65 (46-79)	62 (30-75)	22/30	19/33	simplified FOLFOX-4
Silva, USA,2021	Retrospective, Multi-center	T3 or T4	7694	599 (8%)	60 (12)*	68 (13)*	228/371	3533/3562	NA
Laursen, Denmark,2022	Retrospective, Multi-center	T3 with extramural depth ≥5 mm or T4	1310	179 (14%)	67 (60-73)†	73 (67-80)†	80/99	586/545	NA

nCT, neoadjuvant chemotherapy; pCT, postoperative chemotherapy; RCT, randomized controlled trial; NA, not available.

*Presented with median (standard deviation).

†Presented with mean or mean (standard deviation).

### Primary outcomes

**Figure 2:**
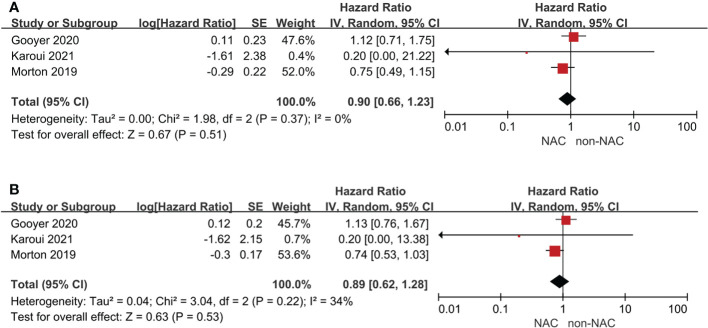
Primary outcomes: **(A)** 3-year overall survival, **(B)** 5-year overall survival.

Four studies reported survival outcomes, but only three could be pooled in meta-analysis. The 3-year OS (HR: 0.90, 95% CI: 0.66-1.23, P = 0.51) and 5-year OS (HR: 0.89, 95% CI: 0.53-1.03, P = 0.53) were comparable with low heterogeneity between the NAC and non-NAC groups **(** **Figure 2** **)**. In addition, Morton (5) reported that no statistically significant difference of cancer-specific mortality or recurrence was observed. Furthermore, the PRODIGE 22 phase II (6) also indicated that disease-free survival, recurrence-free survival, and time to recurrence were all comparable between two groups. Notably, Dehal, et al. (16) founded that no difference of OS was found in the NAC group among patients with T3 (HR: 1.03, 95% CI: 0.85–1.24, P = 0.79) or T4a (HR: 0.97, 95% CI: 0.62-1.53, P = 0.90), but significant benefit could be demonstrated for T4b patients (HR: 0.7, 95% CI: 0.56-0.87, P = 0.002).

### Secondary outcomes

**Figure 3:**
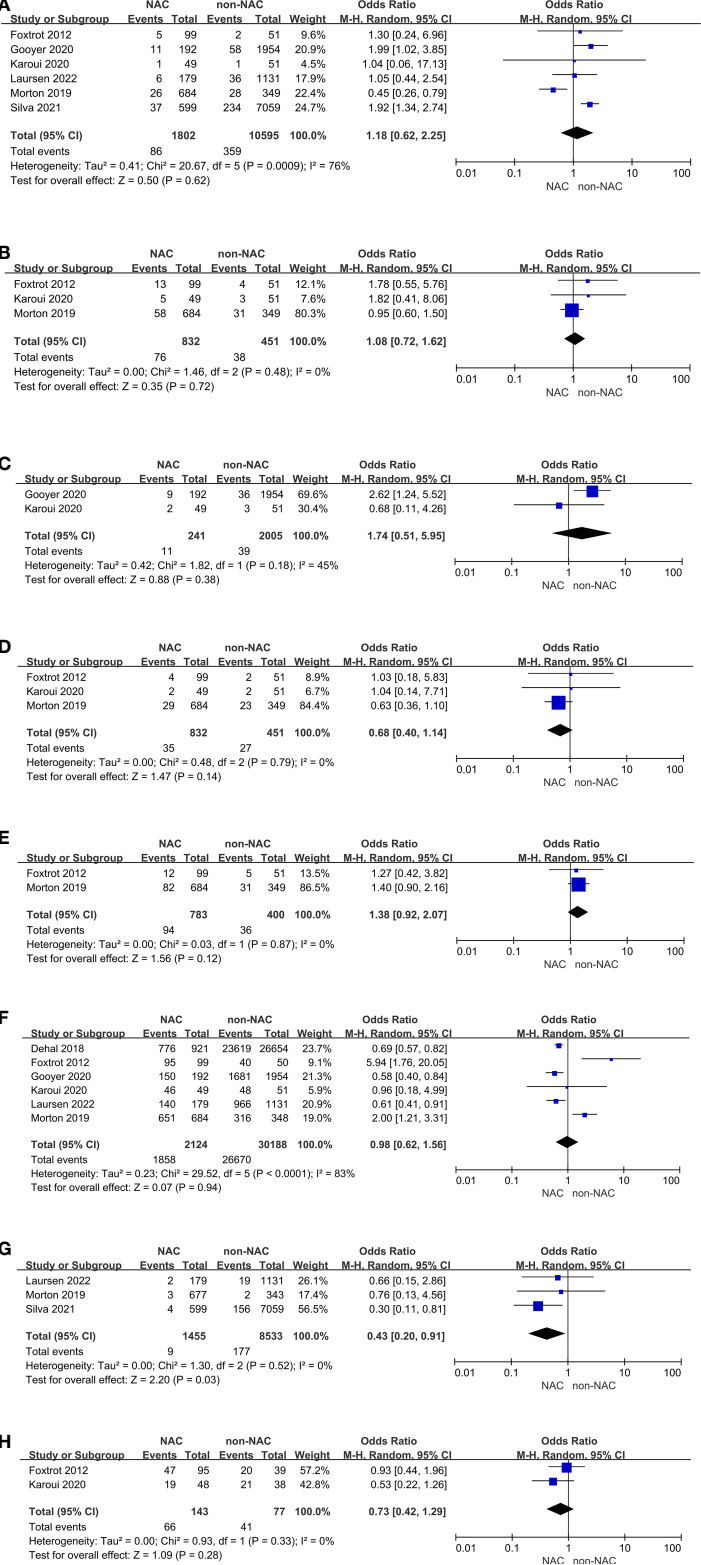
Secondary outcomes: **(A)** Anastomotic leak, **(B)** Wound infection, **(C)** Abscess, **(D)** Re-operation, **(E)** Stoma, **(F)** R0 resection, **(G)** 30-day mortality, **(H)** Adverse events.

There were six studies reporting perioperative complications, adverse events of chemotherapy, or tumor characteristics on pathological examination. No evidence of differences in anastomotic leak (OR: 1.18, 95% CI: 0.62-2.25, P = 0.62), wound infection (OR: 1.08, 95% CI: 0.72-1.62, P = 0.72), abscess (OR: 1.74, 95% CI: 0.51-5.95, P = 0.38), re-operation (OR: 0.68, 95% CI: 0.40-1.14, P = 0.14), stoma (OR: 1.38, 95% CI: 0.92-2.07, P = 0.12), or R0 resection (OR: 0.98, 95% CI: 0.62-1.56, P = 0.94) was found between the NAC and non-NAC groups **(Figures 3A-3F** **)**. Karoui, et al. (15) also reported comparison of postoperative ileus, suggesting that there was no difference between two groups (P = 0.68). However, the NAC group had a significantly lower 30-day mortality than the non-NAC group (OR: 0.43, 95% CI: 0.20-0.91, P = 0.03) **(** **Figure 3G** **)**. On the other hand, Karoui, et al. (15) indicated that no difference was found 60 days after surgery (P = 1.00). Additionally, adverse events of chemotherapy were reported by two RCTs (14, 15), presenting grade 3 or higher adverse events. A meta-analysis was conducted, and no advantage in the NAC group could be found (OR: 0.73, 95% CI: 0.42-1.29, P = 0.28) **(** **Figure 3H** **)**.

### Subgroup analyses

**Figure 4:**
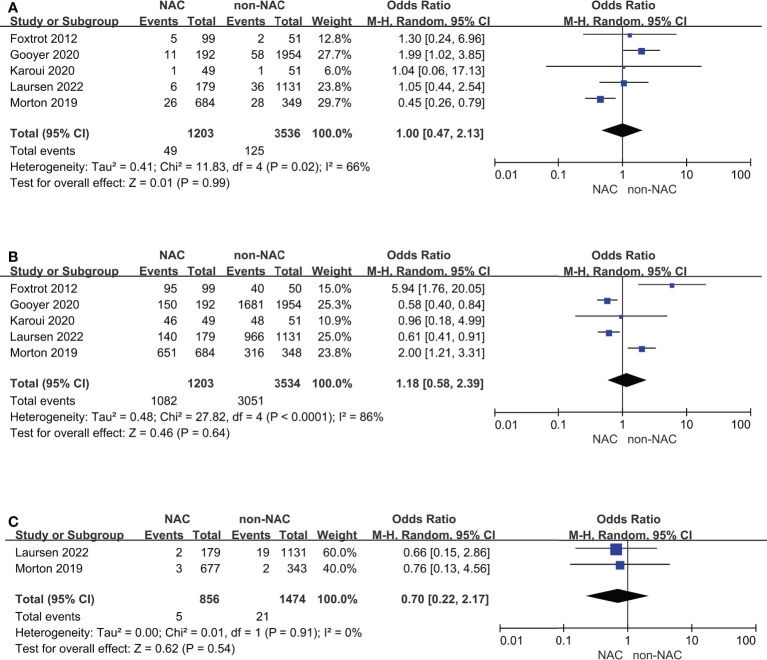
Subgroup analyses for cases diagnosed with T3 with extramural depth ≥ 5 mm or T4: **(A)** Anastomotic leak, **(B)** R0 resection, **(C)** 30-day mortality.

Cases diagnosed with T3 with extramural depth ≥ 5 mm or T4 were separated as a subgroup for further analyses **(** **Figure 4** **)**. Studies reported OS outcomes all met this standard. Anastomotic leak and R0 resection remained comparable and high heterogeneity, but 30-day mortality showed no difference between two groups, which was different from the result of primary outcomes.

**Figure 5:**
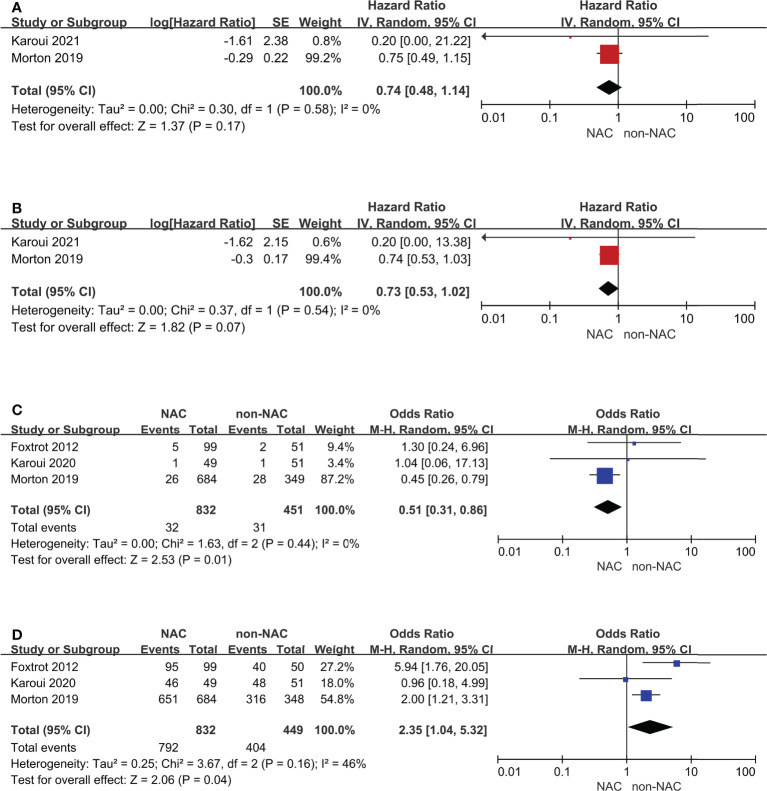
Subgroup analyses for randomized controlled trials: **(A)** 3-year overall survival, **(B)** 5-year overall survival, **(C)** Anastomotic leak, **(D)** R0 resection.

In addition, RCTs were also be selected as a subgroup **(** **Figure 5** **)**. The results of the 3-year and 5-year OS outcomes remained unchanged and had low heterogeneity. Anastomotic leak and R0 resection remained comparable and notably, the heterogeneity (*I* ^2^) decreased from 76% to 0% and from 83% to 46%, respectively.

## Discussion

This systematic review identified 8 available studies with high quality, investigating the effects of NAC for patients with LACC from multiple perspectives. The meta-analysis indicated that NAC was not associated with better survival outcomes, even though it was safe and feasible in perioperative management. It is worth noting that the side effects caused by NAC cannot be ignored. In certain cases, these adverse events even led to the failure of some patients to complete the operation on schedule. On the other hand, there were also patients achieving tumor remission due to preoperative treatment, so they were able to avoid receiving surgery with pain and recovery.

No heterogeneity of the primary outcomes was detected, but the that of anastomotic leak and R0 resection in secondary outcomes was considered high. Sensitivity analysis may be achieved through subgroup analysis. Majority of the studies defined LACC as T3 with extramural depth ≥ 5 mm or T4 determined by computed tomography, and therefore, a subgroup was set according to this criterion. All outcomes showed no evidence supporting the use of NAC in these cases but could not solve the homogeneity. In the subgroup of RCTs, all subgroup remained unchanged, and also, the decrease of *I* ^2^ concerning anastomotic leak and R0 resection helped explain that the source of heterogeneity might be the study design of non-RCTs.

Compared with the two previous studies of meta-analysis (20, 21), more studies were included in the comparison of the feasibility and safety of NAC. Besides, more indicators were evaluated in this study for further assessment, such as abscess, re-operation, stoma, mortality and adverse events of chemotherapy. Regarding the survival outcomes in this study, only two RCTs and one non-RCT were included without a huge quantity of participants yet, which might bring bias. However, due to the addition of new literature in recent years and the elimination of low-quality Chinese documents not recruited in the world-wide database, this study overturned the conclusion that NAC has a survival benefit suggested by the previous meta-analysis (20).

Some patients with LACC cannot be radically cured even with combined organ resection. In addition, neoadjuvant therapy could help judge the biological behavior of tumors. For patients who still have disease progression during neoadjuvant chemotherapy, the significance of surgical resection is very limited. Although the use of NAC for colon cancer has increased significantly over time (16, 17, 22) in certain countries and regions, it is still infrequently as a common practice all over the world yet. According to previous published studies, the major obstacle to promotion might be the uncertainty regarding its potential benefit (23). Further RCTs concerning oncological outcomes is warranted to identify a subgroup of patients who could benefit from neoadjuvant chemotherapy in a perioperative strategy. It is also worth noting that looking at survival benefit only misses the entire point of all the other considerations. There is also potential risk of emergency operation during the period of NAC, especially in those patients with tumor progression or poor condition attributed to chemotherapeutic agents. Fortunately, this meta-analysis and the included RCTs have shown that NAC for LACC is feasible, with acceptable toxicity and perioperative incidence rate. Furthermore, one has to consider the wasted finances of potentially unnecessary preoperative treatment. In order to implement NAC more reasonably and effectively, the key point should be to screen the appropriate subgroup and detect markers available to predict neoadjuvant chemosensitivity (24). Further optimization of clinical staging is essential to accurately select patients who may benefit from neoadjuvant therapy and avoid over treatment of low-risk patients (25). Thus, the use of NAC needs to be assessed in multiple aspects, which was the consideration in formulating this systematic review and meta-analysis.

It should also be noted that neoadjuvant therapy may change the biological characteristics of tumors. For instance, the expression of mismatch repair proteins, commonly MSH6, can change after neoadjuvant therapy (26, 27). Moreover, safety and efficacy of the following surgery may also be influenced because of the implementation of neoadjuvant therapy (28). Novel and personalized prognostic markers also need to be developed regarding patients with history of NAC (29, 30). An initial phase II experience indicates that a large proportion of patients with NAC might be converted to a low-risk state before surgery, thereby eliminating the need for following adjuvant chemotherapy (31). However, as the neoadjuvant treatment of colon cancer is still in the exploratory stage, it has not been recognized by the academic community that patients who have benefited from NAC could be exempt from surgery or postoperative chemotherapy.

Several limitations in this study should be mentioned. One was that not enough studies could be searched and included in the pooled analysis, especially for survival data. Additionally, there is no consensus on the standard of LACC for now yet. Although a majority of studies defined LACC as T3 with extramural depth ≥ 5 mm or T4, few research have been able to accurately include these cases except RCTs. Recent studies have shown that when planning NAC for LACC, preoperative computed tomography scan with around 60% consistent with pathologic results in T stage or extramural invasion may overestimate the clinical stage and lead to inappropriate treatment (32–34). All of the above limitations have contributed to the heterogeneity among the included studies. Nevertheless, this research is of significance in the field of LACC, timely providing high-level evidence for clinical practice based on the available evidence.

## Conclusion

Overall, NAC is safe and feasible for patients with LACC, but no significant survival benefit could be demonstrated. The application of NAC still needs to be prudent in the near future until significant evidence supporting the oncological outcomes is presented.

## Data availability statement

The original contributions presented in the study are included in the article/**Supplementary Material**. Further inquiries can be directed to the corresponding authors.

## Author contributions

ZYL, ZL, QY, and WW contributed to conception and design of the study. JF, DX, and HL performed the paper searching. ZYL and GM performed the statistical analysis. ZYL wrote the first draft of the manuscript. All authors contributed to the article and approved the submitted version.

## Conflict of interest

The authors declare that the research was conducted in the absence of any commercial or financial relationships that could be construed as a potential conflict of interest.

## Publisher’s note

All claims expressed in this article are solely those of the authors and do not necessarily represent those of their affiliated organizations, or those of the publisher, the editors and the reviewers. Any product that may be evaluated in this article, or claim that may be made by its manufacturer, is not guaranteed or endorsed by the publisher.
