# Supplementary material for: The role of neoadjuvant chemotherapy in patients with locally advanced colon cancer: A systematic review and meta-analysis
Source: Front Oncol. 2022 Oct 11;12:1024345. doi: 10.3389/fonc.2022.1024345 (PMC9600337; doi:10.3389/fonc.2022.1024345)
Supplement: Supplementary file 1 [file DataSheet_1.docx]

**Supplementary Table 1: Database searched from PubMed (until April 2022)**

| **#** | **Searches** |
| --- | --- |
| 1 | "Colonic Neoplasms"[Mesh] |
| 2 | "colonic neoplasm"[Title/Abstract] OR "neoplasm colonic"[Title/Abstract] OR "neoplasms colonic"[Title/Abstract] OR "colon neoplasms"[Title/Abstract] OR "colon neoplasm"[Title/Abstract] OR "neoplasm colon"[Title/Abstract] OR "neoplasms colon"[Title/Abstract] OR "cancer of colon"[Title/Abstract] OR "colon cancers"[Title/Abstract] OR "colon cancer"[Title/Abstract] OR "cancer colon"[Title/Abstract] OR "cancers colon"[Title/Abstract] OR "cancer of the colon"[Title/Abstract] OR "colonic cancer"[Title/Abstract] OR "cancer colonic"[Title/Abstract] OR "cancers colonic"[Title/Abstract] OR "colonic cancers"[Title/Abstract] OR "colon adenocarcinoma"[Title/Abstract] OR "adenocarcinoma colon"[Title/Abstract] OR "adenocarcinomas colon"[Title/Abstract] OR "colon adenocarcinomas"[Title/Abstract] |
| 3 | 1 OR 2 |
| 4 | "Neoadjuvant Therapy"[Mesh] |
| 5 | preoperative chemotherapy[Title/Abstract] |
| 6 | (((((((((((((((((((Neoadjuvant Therapies[Title/Abstract]) OR (Therapy, Neoadjuvant[Title/Abstract])) OR (Neoadjuvant Treatment[Title/Abstract])) OR (Neoadjuvant Treatments[Title/Abstract])) OR (Treatment, Neoadjuvant[Title/Abstract])) OR (Neoadjuvant Systemic Therapy[Title/Abstract])) OR (Neoadjuvant Systemic Therapies[Title/Abstract])) OR (Systemic Therapy, Neoadjuvant[Title/Abstract])) OR (Therapy, Neoadjuvant Systemic[Title/Abstract])) OR (Neoadjuvant Systemic Treatment[Title/Abstract])) OR (Neoadjuvant Systemic Treatments[Title/Abstract])) OR (Systemic Treatment, Neoadjuvant[Title/Abstract])) OR (Treatment, Neoadjuvant Systemic[Title/Abstract])) OR (Neoadjuvant Chemotherapy[Title/Abstract])) OR (Chemotherapy, Neoadjuvant[Title/Abstract])) OR (Neoadjuvant Chemotherapies[Title/Abstract])) OR (Neoadjuvant Chemotherapy Treatment[Title/Abstract])) OR (Chemotherapy Treatment, Neoadjuvant[Title/Abstract])) OR (Neoadjuvant Chemotherapy Treatments[Title/Abstract])) OR (Treatment, Neoadjuvant Chemotherapy[Title/Abstract]) |
| 7 | 4 OR 5 OR 6 |
| 8 | 3 AND 7 |
| **Total hits** | **624** |

**Supplementary Table 2: Database searched from Embase (until April 2022)**

| **#** | **Searches** |
| --- | --- |
| 1 | 'colon cancer'/exp |
| 2 | ‘colonic neoplasm’:ti,ab OR ‘neoplasm colonic’:ti,ab OR ‘neoplasms colonic’:ti,ab OR ‘colon neoplasms’:ti,ab OR ‘colon neoplasm’:ti,ab OR ‘neoplasm colon’:ti,ab OR ‘neoplasms colon’:ti,ab OR ‘cancer of colon’:ti,ab OR ‘colon cancers’:ti,ab OR ‘colon cancer’:ti,ab OR ‘cancer colon’:ti,ab OR ‘cancers colon’:ti,ab OR ‘cancer of the colon’:ti,ab OR ‘colonic cancer’:ti,ab OR ‘cancer colonic’:ti,ab OR ‘cancers colonic’:ti,ab OR ‘colonic cancers’:ti,ab OR ‘colon adenocarcinoma’:ti,ab OR ‘adenocarcinoma colon’:ti,ab OR ‘adenocarcinomas colon’:ti,ab OR ‘colon adenocarcinomas’:ti,ab |
| 3 | 1 OR 2 |
| 4 | 'neoadjuvant chemotherapy'/exp |
| 5 | 'preoperative chemotherapy'/exp |
| 6 | 'neoadjuvant therapies':ti,ab OR 'therapy, neoadjuvant':ti,ab OR 'neoadjuvant treatment':ti,ab OR 'neoadjuvant treatments':ti,ab OR 'treatment, neoadjuvant':ti,ab OR 'neoadjuvant systemic therapy':ti,ab OR 'neoadjuvant systemic therapies':ti,ab OR 'systemic therapy, neoadjuvant':ti,ab OR 'therapy, neoadjuvant systemic':ti,ab OR 'neoadjuvant systemic treatment':ti,ab OR 'neoadjuvant systemic treatments':ti,ab OR 'systemic treatment, neoadjuvant':ti,ab OR 'treatment, neoadjuvant systemic':ti,ab OR 'neoadjuvant chemotherapy':ti,ab OR 'chemotherapy, neoadjuvant':ti,ab OR 'neoadjuvant chemotherapies':ti,ab OR 'neoadjuvant chemotherapy treatment':ti,ab OR 'chemotherapy treatment, neoadjuvant':ti,ab OR 'neoadjuvant chemotherapy treatments':ti,ab OR 'treatment, neoadjuvant chemotherapy':ti,ab |
| 7 | 4 OR 5 OR 6 |
| 8 | 3 AND 7 |
| **Total hits** | **2368** |

**Supplementary Table 3: Database searched from Web of Science (until April 2022)**

| **#** | **Searches** |
| --- | --- |
| 1 | (((((((((((((((((((((TS=(Colonic Neoplasms)) OR TS=(Colonic Neoplasm)) OR TS=(Neoplasm, Colonic)) OR TS=(Neoplasms, Colonic)) OR TS=(Colon Neoplasms)) OR TS=(Colon Neoplasm)) OR TS=(Neoplasm, Colon)) OR TS=(Neoplasms, Colon)) OR TS=(Cancer of Colon)) OR TS=(Colon Cancers)) OR TS=(Colon Cancer)) OR TS=(Cancer, Colon)) OR TS=(Cancers, Colon)) OR TS=(Cancer of the Colon)) OR TS=(Colonic Cancer)) OR TS=(Cancer, Colonic)) OR TS=(Cancers, Colonic)) OR TS=(Colonic Cancers)) OR TS=(Colon Adenocarcinoma)) OR TS=(Adenocarcinoma, Colon)) OR TS=(Adenocarcinomas, Colon)) OR TS=(Colon Adenocarcinomas) |
| 2 | ((((((((((((((((((((TS=(Preoperative Chemotherapy)) OR TS=(Neoadjuvant Therapies)) OR TS=(Therapy, Neoadjuvant)) OR TS=(Neoadjuvant Treatment)) OR TS=(Neoadjuvant Treatments)) OR TS=(Treatment, Neoadjuvant)) OR TS=(Neoadjuvant Systemic Therapy)) OR TS=(Neoadjuvant Systemic Therapies)) OR TS=(Systemic Therapy, Neoadjuvant)) OR TS=(Therapy, Neoadjuvant Systemic)) OR TS=(Neoadjuvant Systemic Treatment)) OR TS=(Neoadjuvant Systemic Treatments)) OR TS=(Systemic Treatment, Neoadjuvant)) OR TS=(Treatment, Neoadjuvant Systemic)) OR TS=(Neoadjuvant Chemotherapy)) OR TS=(Chemotherapy, Neoadjuvant)) OR TS=(Neoadjuvant Chemotherapies)) OR TS=(Neoadjuvant Chemotherapy Treatment)) OR TS=(Chemotherapy Treatment, Neoadjuvant)) OR TS=(Neoadjuvant Chemotherapy Treatments)) OR TS=(Treatment, Neoadjuvant Chemotherapy) |
| 3 | 1 AND 2 |
| **Total hits** | **2057** |

**Supplementary Table 4: Database searched from Cochrane Library (until April 2022)**

| **#** | **Searches** |
| --- | --- |
| 1 | MeSH descriptor: [Colonic Neoplasms] explode all trees |
| 2 | (Colonic Neoplasm):ti,ab,kw OR (Neoplasm, Colonic):ti,ab,kw OR (Neoplasms, Colonic):ti,ab,kw OR (Colon Neoplasms):ti,ab,kw OR (Colon Neoplasm):ti,ab,kw OR (Neoplasm, Colon):ti,ab,kw OR (Neoplasms, Colon):ti,ab,kw OR (Cancer of Colon):ti,ab,kw OR (Colon Cancers):ti,ab,kw OR (Colon Cancer):ti,ab,kw OR (Cancer, Colon):ti,ab,kw OR (Cancers, Colon):ti,ab,kw OR (Cancer of the Colon):ti,ab,kw OR (Colonic Cancer):ti,ab,kw OR (Cancer, Colonic):ti,ab,kw OR (Cancers, Colonic):ti,ab,kw OR (Colonic Cancers):ti,ab,kw OR (Colon Adenocarcinoma):ti,ab,kw OR (Adenocarcinoma, Colon):ti,ab,kw OR (Adenocarcinomas, Colon):ti,ab,kw OR (Colon Adenocarcinomas):ti,ab,kw |
| 3 | 1 OR 2 |
| 4 | MeSH descriptor: [Neoadjuvant Therapy] explode all trees |
| 5 | (Neoadjuvant Therapies):ti,ab,kw OR (Therapy, Neoadjuvant):ti,ab,kw OR (Neoadjuvant Treatment):ti,ab,kw OR (Neoadjuvant Treatments):ti,ab,kw OR (Treatment, Neoadjuvant):ti,ab,kw OR (Neoadjuvant Systemic Therapy):ti,ab,kw OR (Neoadjuvant Systemic Therapies):ti,ab,kw OR (Systemic Therapy, Neoadjuvant):ti,ab,kw OR (Therapy, Neoadjuvant Systemic):ti,ab,kw OR (Neoadjuvant Systemic Treatment):ti,ab,kw OR (Neoadjuvant Systemic Treatments):ti,ab,kw OR (Systemic Treatment, Neoadjuvant):ti,ab,kw OR (Treatment, Neoadjuvant Systemic):ti,ab,kw OR (Neoadjuvant Chemotherapy):ti,ab,kw OR (Chemotherapy, Neoadjuvant):ti,ab,kw OR (Neoadjuvant Chemotherapies):ti,ab,kw OR (Neoadjuvant Chemotherapy Treatment):ti,ab,kw OR (Chemotherapy Treatment, Neoadjuvant):ti,ab,kw OR (Neoadjuvant Chemotherapy Treatments):ti,ab,kw OR (Treatment, Neoadjuvant Chemotherapy):ti,ab,kw |
| 6 | 4 OR 5 |
| 7 | 3 AND 6 |
| **Total hits** | **209** |

**Supplementary Table 5: Full-text articles excluded**

| **Reasons** | **Number of studies** | **References** |
| --- | --- | --- |
| Single arm | 10 | (1-10) |
| Cases with rectal cancer | 2 | (11, 12) |
| Incomplete data | 8 | (13-20) |
| Without outcomes of interest | 1 | (21) |
| Only protocol | 11 | (22-32) |

**References of excluded studies**

1. Arredondo J, Pastor C, Baixauli J, Rodriguez J, Gonzalez I, Vigil C, et al. Preliminary outcome of a treatment strategy based on perioperative chemotherapy and surgery in patients with locally advanced colon cancer. COLORECTAL DISEASE. 2013;15(5):552-7.

2. Arredondo J, González I, Baixauli J, Martínez P, Rodríguez J, Pastor C, et al. Tumor response assessment in locally advanced colon cancer after neoadjuvant chemotherapy. Journal of gastrointestinal oncology. 2014;5(2):104-11.

3. Fusco JP, Martin RP, Aldaz A, Baixauli J, Subtil JC, Hernandez-Lizoain JL, et al. A retrospective analysis of preoperative FOLFOX chemotherapy for locally advanced colon cancer patients with pharmacokinetic-guided dose adjustements of 5-FU: Preliminary results. Annals of Oncology. 2014;25:ii83-ii4.

4. Rodriguez J, Romano PM, Aldaz A, Chopitea A, Baixauli J, Subtil JC, et al. Preliminary results of preoperative FOLFOX chemotherapy for locally advanced colon cancer patients with therapeutic drug monitoring of 5-FU. Journal of Clinical Oncology. 2014;32(3).

5. Jakobsen A, Andersen F, Fischer A, Jensen LH, Jørgensen JCR, Larsen O, et al. Neoadjuvant chemotherapy in locally advanced colon cancer. A phase II trial. Acta Oncologica. 2015;54(10):1747-53.

6. Aisu N, Yoshida Y, Komono A, Yamada T, Kojima D, Mera T, et al. Perioperative chemotherapy with S-1 plus oxaliplatin (SOX) for stage III colorectal cancer patients. Journal of Clinical Oncology. 2016;34(4).

7. Liu FQ, Yang L, Wu YC, Li C, Zhao J, Keranmu A, et al. CapOX as neoadjuvant chemotherapy for locally advanced operable colon cancer patients: a prospective single-arm phase II trial. CHINESE JOURNAL OF CANCER RESEARCH. 2016;28(6):589-97.

8. Yang L, Liu FQ, Huang D, Xu JY, Huang L, Xu Y. Neoadjuvant chemotherapy with XELOX regimen for locally advanced operable colon cancer patients: a prospective phase II trial (NCT02415829). JOURNAL OF CLINICAL ONCOLOGY. 2016;34(15).

9. Arredondo J, Baixauli J, Pastor C, Chopitea A, Sola JJ, González I, et al. Mid-term oncologic outcome of a novel approach for locally advanced colon cancer with neoadjuvant chemotherapy and surgery. Clinical & translational oncology : official publication of the Federation of Spanish Oncology Societies and of the National Cancer Institute of Mexico. 2017;19(3):379-85.

10. Nct. Perioperative Versus Postoperative CapOX Chemotherapy for Locally Advanced Colon Cancer. <https://clinicaltrialsgov/show/NCT03125980>. 2017.

11. Ceniceros L, Pastor C, Sanchez-Justicia C, Arean C, Baixauli J, Chopitea A, et al. Neoadjuvant chemotherapy for locally advanced colon cancer patients: Long-term results from a single institutional experience. JOURNAL OF CLINICAL ONCOLOGY. 2021;39(15).

12. Wei S, Xi J, Cao S, Li T, Xu J, Li W, et al. Laparoscopic radical resection combined with neoadjuvant chemotherapy in treatment of colorectal cancer: Clinical efficacy and postoperative complications. American Journal of Translational Research. 2021;13(12):13974-80.

13. Lv Y, Dai G. The efficacy of neoadjuvant chemotherapy in patients with advanced colon cancer. Annals of Oncology. 2016;27:ix59.

14. Artinyan A, Seiser N, Zhu R, Stettler I, Shen W, Shirinian M, et al. Preoperative chemotherapy is associated with worseoverall survival in operable colon cancer. Diseases of the Colon and Rectum. 2020;63(6):e153-e4.

15. Hussein MK, Al-Quda G, Keane CA, Marar O, Blebea J. Trends and Outcomes of Using Neoadjuvant Systemic Therapy for Clinical T4b-M0 Colon Cancer in the US: An Analysis of the National Cancer Database. Journal of the American College of Surgeons. 2021;233(5):S64-S5.

16. Glasbey J, Morton D. Risk of bowel obstruction in patients undergoing neoadjuvant chemotherapy for high-risk colon cancer: A nested case-control matched analysis of an international, multi-centre, randomised trial (FOxTROT). Colorectal Disease. 2022;24(SUPPL 1):97-8.

17. Verstegen M, Gooyer J, T Lam-Boer J, Radema S, Ten Tije A, Elferink M, et al. Locally advanced colon cancer and the use of neo-adjuvant chemotherapy in the Netherlands. European Journal of Cancer. 2015;51:S382.

18. Verstegen M, Gooyer JM, T'Lam-Boer J, Radema S, Elferink M, Ten Tije A, et al. The use of neo-adjuvant chemotherapy for locally advanced colon cancer in the Netherlands. Annals of Oncology. 2015;26:iv63.

19. dos Santos LV, Faria TMV, Lima ABC, Abdalla KC, de Moraes ED, Cruz MR, et al. Timing of adjuvant chemotherapy in colorectal cancer. COLORECTAL DISEASE. 2016;18(9):871-6.

20. Verstegen MG, De Gooyer JM, T'Lam-Boer J, Radema SA, Verhoeven RHA, Verhoef C, et al. Downstaging effects of neoadjuvant chemotherapy in locally advanced colon cancer. Colorectal Disease. 2018;20:93.

21. Murakami PK, West NP, Ide R, Richman SD, Magill L, Gray R, et al. The relationship between tumour immune profile and response to folfox-based pre-operative chemotherapy in the international phase iii foxtrot trial. Journal of Pathology. 2019;249:S37.

22. Nct. Neoadjuvant Chemotherapy Versus Standard Treatment in Patients With Locally Advanced Colon Cancer. <https://clinicaltrialsgov/show/NCT01918527>. 2013.

23. Euctr SE. Neoadjuvant chemotherapy versus standard treatment in patients with locally advanced colon cancer. <https://trialsearchwhoint/Trial2aspx?TrialID=EUCTR2013-002363-26-SE>. 2016.

24. Nct. Laparoscopic Surgery VS Laparoscopic Surgery + Neoadjuvant Chemotherapy for T4 Tumor of the Colon Cancer. <https://clinicaltrialsgov/show/NCT02777437>. 2016.

25. Nct. Neoadjuvant Chemotherapy for the Treatment of Resectable Locally Advanced Colon Cancer. <https://clinicaltrialsgov/show/NCT02882269>. 2016.

26. Nct. Neoadjuvant FOLFOX Chemotherapy for Patients With Locally Advanced Colon Cancer. <https://clinicaltrialsgov/show/NCT03426904>. 2018.

27. Liu F, Tong T, Huang D, Yuan W, Li D, Lin J, et al. CapeOX perioperative chemotherapy versus postoperative chemotherapy for locally advanced resectable colon cancer: protocol for a two-period randomised controlled phase III trial. BMJ open. 2019;9(1).

28. Nct. Analysis of the Effectiveness of Neoadjuvant Chemotherapy in the Treatment of Colon Cancer Locally Advanced (ELECLA). <https://clinicaltrialsgov/show/NCT04188158>. 2019.

29. Vermorken J, Cervantes A, Morsing P, Johansson K, Andersson T, Lindland Roest N, et al. A randomized, multicenter, open-label controlled phase 2 trial of Foxy-5 as neoadjuvant therapy in patients with WNT5A negative colon cancer. Annals of oncology. 2019;30:AA36‐.

30. Isrctn. A trial assessing preoperative chemotherapy in patients with locally advanced but operable colon cancer. <https://trialsearchwhoint/Trial2aspx?TrialID=ISRCTN83842641>. 2021.

31. Tong Z, Lu S, Dai X, Cheng X, Bao X, Zhu X, et al. Camrelizumab and apatinib combined with chemotherapy (mFOLFOX6) as neoadjuvant therapy for locally advanced right-sided colon cancer (ambition). Journal for ImmunoTherapy of Cancer. 2021;9(SUPPL 2):A443‐.

32. Nct. Neoadjuvant FOLFOXIRI Versus Immediate Surgery for Stage II and III Colon Cancers. <https://clinicaltrialsgov/show/NCT05194878>. 2022.

**Supplementary Table 6: Methodological quality and risk of bias**

| **Study** | **Modified Jadad Score** | **New Castle Ottawa (NOS)** | | | |
| --- | --- | --- | --- | --- | --- |
|  |  | **Selection**  **(0-4)** | **Comparability**  **(0-2)** | **Outcome**  **(0-3)** | **Total**  **(0-9)** |
| Foxtrot,  UK,2012 | 5 | / | / | / | / |
| Dehal,  USA,2018 | / | 2 | 2 | 3 | 7 |
| Morton,  UK,2019 | 5 | / | / | / | / |
| Gooyer,  NLD,2020 | / | 2 | 2 | 3 | 7 |
| Karoui,  France,2020 | 4 | / | / | / | / |
| Karoui,  France,2021 | 4 | / | / | / | / |
| Silva,  USA,2021 | / | 2 | 2 | 3 | 7 |
| Laursen,  Denmark,2022 | / | 2 | 2 | 3 | 7 |
